# The Role of Serum Cytokines in the Pathogenesis of Hepatic Osteodystrophy in Male Cirrhotic Patients

**DOI:** 10.1155/2012/425079

**Published:** 2012-10-14

**Authors:** Ali Riza Soylu, Cengiz Tuglu, Ender Arikan, Tarkan Yetisyigit, Hakan Kunduracılar, Ibrahim Hakki Koker, Gulbin Unsal, Ahmet Huseyin Tezel, Hasan Umit, Sakir Berkarda

**Affiliations:** ^1^Department of Gastroenterology, Faculty of Medicine, Trakya University, 22030 Edirne, Turkey; ^2^Department of Psychiatry, Faculty of Medicine, Trakya University, 22030 Edirne, Turkey; ^3^Department of Endocrinology and Metabolism, Faculty of Medicine, Acıbadem University, 34848 Istanbul, Turkey; ^4^Department of Oncology, Ankara Oncology Hospital, 06200 Ankara, Turkey; ^5^Health Services Vocational School, Trakya University, 22030 Edirne, Turkey; ^6^Department of Nuclear Medicine, Faculty of Medicine, Trakya University, 22030 Edirne, Turkey

## Abstract

*Objective*. In this study, we aimed to investigate the possible role of serum cytokines in the development of hepatic osteodystrophy. *Matherial and Methods*. 44 consecutive male cirrhotic patients (17 alcoholic, 20 hepatitis B, 7 hepatitis C), 15 age- and sex-matched chronic alcoholics without liver disease, and 17 age- and sex-matched healthy controls were included in the study during one year period. Bone mineral density was measured by dual X-ray absorptiometry in the lumbar vertebrate and femoral neck. Serum interleukin levels were measured by ELISA method. *Results*. Although osteopenia frequency between our cirrhotic patients was 20%, there was no difference in T-scores among the controls and other groups. Serum interleukin-1, interleukin-8, and tumor necrosis factor-alpha levels were not different between all groups. Serum interleukin-2 and interleukin-6 levels were higher in the cirrhotics than controls (*P* < 0.001). However, there were no significant difference between osteopenic and nonosteopenic cirrhotics. *Conclusion*. According to the results of the study in this small population of 44 male cirrhotic patients, frequency of hepatic osteopenia is small and serum interleukins 1, 2, 6, 8, and tumor necrosis factor-alpha may not play a role in the pathogenesis of hepatic osteodystrophy. Further studies in which large number of patients involved are necessary in this field.

## 1. Introduction

Osteoporosis prevalence studies conducted among patients with chronic liver disease were reported to be high in a range of 8.6% and 53% according to patient selection and diagnostic criteria [1–5]. However, the pathogenesis of hepatic osteoporosis is not well understood. Potential inciting factors include insulin-like growth factor-1 (IGF-1) deficiency, hyperbilirubinemia, alcoholism, vitamin D deficiency, vitamin D receptor genotype polymorphism, immunosuppressive agents used before and following liver transplantation, and hypogonadism (estrogen and testosterone deficiency) [6].

In studies conducted on healthy postmenopausal women, estrogen deficiency was demonstrated to cause osteoporosis by locally stimulating interleukin-1 (IL-1), IL-6, and tumor necrosis factor-*α* (TNF-*α*) synthesis [7]. Interleukin-1 and TNF-*α* have been shown to be potent bone resorbing agents stimulating osteoclastic activity [8–12]. On the other hand, chronic liver diseases are among a group of immune dysregulation disorders associated with chronic inflammation and tissue damage that are frequently associated with increased serum levels of cytokines such as IL-1, IL-6, and TNF-*α* [13–15]. However, it is unclear whether these high levels of serum cytokines accompanying chronic inflammation of the liver observed in patients with cirrhosis leads to bone loss. It was demonstrated that high levels of soluble TNF receptors (sTNFR) in serum were inversely proportional to bone mineral density in patients with viral cirrhosis [16]. In another study, bone loss due to chronic alcohol consumption in mice was reported to be associated with IL-6, and that bone loss was not demonstrated in IL-6^−/−^ knockout mice [17].

The aim of this controlled study was to identify the serum levels of IL-1, IL-2, IL-6, IL-8, and TNF-*α* in patients with viral and alcoholic cirrhosis and to investigate the relationship of these cytokines to bone loss in male cirrhotic patients. In order to clarify the role of cytokines on bone loss, the other probable contributors of bone loss including calcitonin, parathormone, 25-OH vitamin D, testosterone, and osteocalcin were also investigated.

## 2. Methods

### 2.1. Patients

A total of 44 consecutive male patients, who developed liver cirrhosis associated with hepatitis B virus, hepatitis C virus, or alcohol abuse and were followed up at the Gastroenterology Clinic of Trakya University Hospital, were included in the study. In cirrhosis group, 27 patients had viral etiology, while 17 patients had alcoholic cirrhosis. As a control group for the alcohol abusing cirrhotics, a total of 15 age-matched alcoholic male patients who have been consuming ≥60 g/day of ethanol for at least eight years without any damage to liver function and were followed up at the Psychiatry Clinic of the same hospital were included. These patients were followed up for a period of one year. In the healthy control group, 17 individuals, whose ages and gender matched with the study criteria, were enrolled.

The diagnosis of liver cirrhosis was made using clinical, radiological, and biochemical or coagulation parameters and the definite diagnosis was made through liver biopsy in patients with normal platelet count and function. Patients at the age of 55 and above who were candidates to develop spontaneous osteodystrophy and osteoporosis in the future, patients with hepatocellular carcinoma, and patients with significant cardiac, pulmonary, renal failure, or serious infections were excluded from the study. Clinical characteristics of the study group are shown in Table 1. Fasting blood samples were collected very early in the morning and stored under −86°C in the deep-freezer.

Approval for this study was obtained from the Trakya University Local Ethics Committee.

### 2.2. Basic Parameters

The medical history of all patients and controls that were included in the study were obtained, and results of the physical examination and laboratory evaluations, and data concerning alcohol, caffeine containing drinks, and dairy product consumption were recorded. Height and weight of the patients were also determined and the body mass index (BMI) was calculated.

### 2.3. Liver Disease

The serum aspartate aminotransferase (AST), alanine aminotransferase (ALT), alkaline phosphatase (ALP), gamma-glutamyl transferase (GGT), bilirubin (total and conjugated), and the serum albumin levels were measured by Olympus AU 800 biochemical automated analyzer.

### 2.4. Bone Density Analysis

Bone density of all patients was measured using dual-energy X-ray absorptiometry of the lumbar vertebrae and femoral neck. Measurements of the femoral neck and lumbar 1, 2, and 3 vertebrae were evaluated. Diagnoses of osteoporosis and osteopenia were performed according to the WHO criteria, with the diagnosis of osteoporosis defined as a mean T score of −2.5 SD and below, whereas diagnosis of osteopenia was defined as a T score between −1 and −2.5 SD [18].

### 2.5. Bone Metabolism and Hormones

The serum 25-OH vitamin D level was measured using the ELISA method (immunodiagnostic AG, Bensheim, Germany), whereas parathormone, testosterone, free T3, free T4, TSH, and osteocalcin levels were evaluated using the chemical immunoassay method.

### 2.6. Serum Cytokines

The serum IL-1B, IL-2R, IL-6, IL-8, and TNF-*α* levels were measured by the ELISA method (BioSource International, Inc., California, USA).

### 2.7. Biostatistics

Data are presented as mean ± standard deviation. Continuous variable normality was determined by Shapiro-Wilk's test. The one-way ANOVA test was used to analyze the statistical difference between groups for data with normal distribution of all variables, whereas Kruskal-Wallis test was used for the analysis of data with nonnormal distribution. Kruskal-Wallis posthoc multiple comparison test was used for data with normal distribution, whereas the Bonferroni's post hoc multiple comparison test was used for data with nonnormal distribution. A *P* value <0.05 was considered as statistically significant.

## 3. Results

### 3.1. Basic Parameters of Liver Disease and Bone Metabolism and Hormones

There was no significant difference between the groups with regard to age, height, weight, and consumption of dairy products (Table 1). However, cigarette smoking was found to be statistically lower in the control group compared to the patients with cirrhosis and alcoholic controls (*P* < 0.001). On the other hand, caffeine consumption was found to be significantly lower in the group with alcoholic cirrhosis compared to the other groups (*P* < 0.001). Alkaline phosphatase and GGT levels were particularly higher in patients with alcoholic cirrhosis. The serum calcium level was found to be decreased in the cirrhotic group due to low serum albumin levels; however, the corrected calcium levels were within normal range. The serum phosphorus, 24-hour urinary calcium, and phosphorus levels were not found to be different between the groups. There was no difference between the groups with regards to serum parathormone, calcitonin, and 25-OH vitamin D levels (*P* > 0.05). The serum osteocalcin levels in cirrhotic patients was found to be significantly decreased when compared to the control group (*P* < 0.001) (Table 2). Serum total testosterone levels were shown to be high in alcoholic controls only when compared to the other groups (*P* < 0.01) (Table 2). Serum TSH levels were found to be decreased in both cirrhotic patient groups compared to the healthy and alcoholic controls (*P* < 0.05). Serum free T3 levels were lower, *n* alcoholic cirrhotics compared to all other groups (*P* < 0.05).

### 3.2. Bone Mineral Density

Comparison of patients with cirrhosis and the control group using the Kruskal-Wallis test demonstrated that there was no statistically significant difference in the T scores (Table 2). Although there were 5 patients (29%) with osteopenia in the control group, nine patients (20%) (4 hepatitis B, 1 hepatitis C, and 4 alcoholic cirrhosis patients) with a T score of between −1 and −2.5 in the cirrhosis group, and five individuals (33%) in the alcoholic control group were observed to have osteopenia. Only one patient in the cirrhotic group (alcoholic cirrhosis) was found to have a T score below −2.5 (prevalence of osteoporosis was 1.9%), whereas in the alcoholic control group, two individuals had a T score below −2.5 (prevalence of osteoporosis was 13%).

### 3.3. Serum Cytokines

There was no significant difference between the groups with regard to serum IL-1, IL-8, and TNF-alpha levels (Table 2). Only serum IL-2 and IL-6 levels were found to be significantly higher in cirrhotic patients (*P* < 0.001). However, no significant difference was detected in serum IL-2 and IL-6 levels between cirrhotic patients with and without osteopenia. 

## 4. Discussion

In our study, osteopenia frequencies in cirrhotics (20%) and chronic alcoholics without liver disease (29%) were similar to controls (33%). Thus in our male cirrhotic patients liver disease did not predispose osteopenia. We suggest that the reason for the low frequency of osteopenia in the cirrhotic group was probably due to the fact that the study population consisted completely of men and the relatively low mean age of the study population, taking into consideration the late onset of the tendency for osteoporosis in men [19]. In the present study, our male cirrhotic patients and chronic alcoholics without liver disease have normal testosterone levels in contrast to the usual expected decrease in cirrhotics [20]. Although we did not measure, it is well known that estrogen levels in male patients with cirrhosis is increased due to reduced metabolism from the liver and increased conversion of testosterone and androstenedione to estrone and estradiol in the peripheral tissues [21]. The possible excess estrogen in our male patients with cirrhosis in combination with proven normal serum testosterone levels may protect our cirrhotic patients from bone loss. Another factor which might have affected results of our study was the fact that healthy controls had a lesser habit of cigarette smoking and alcoholic cirrhotic patients consumed less caffeine containing food. Cigarette smoking probably increases the tendency for the development of osteoporosis, associated with its common use in alcoholics. 

In our study, no significant difference was observed between cirrhotic patients with osteopenia and without osteopenia, with regards to serum cytokine levels. The normal levels of serum IL-1, IL-8, and TNF-*α* in our study in cirrhotic patients with osteopenia compared to the controls indicated that these cytokines do not play a role in the pathogenesis of hepatic osteodystrophy. To our knowledge, the relationship of serum IL-8 to hepatic osteopenia has not been previously investigated. The current study is the first to report a lack of significant association between serum IL-8 and bone mineral density. The high IL-2 and IL-6 levels in our cirrhotic patients suggest that these cytokine may play a role in the pathogenesis of hepatic osteoporosis. However, no significant difference was demonstrated between cirrhotic patients with and without osteopenia with regards to IL-2 and IL-6 levels. A previously conducted *in vitro* study was also demonstrated that IL-2 had no effect on osteoclasts [22]. 

The serum levels of IL-6, IL-8, and IL-18, were reported to be increased secondary to systemic endotoxemia, in patients with alcoholic liver disease and steatohepatitis [23]. The serum IL-6 levels of our alcoholic cirrhotic patient group were also found to be increased compared to controls and chronic alcohol user. However, there was no significant difference between alcoholic cirrhotic patients with and without osteopenia. On the other hand, chronic inflammation may show some difference in the degree of activity during the course of time. This means that an osteopenic cirrhotic patient may present with severe active hepatitis for many years and then demonstrate inactive cirrhosis during the course of the study. Serum cytokines evaluated during this period may show normal values, while bone mineral loss may be prominent despite normal cytokine levels. Cross-sectional evaluation of serum cytokine levels may have probably been obscuring a possible relationship. 

In accordance with our findings, another recent study by Mitchell et al. failed to detect an association between serum cytokines IL-6 and TNF*α* and bone mineral density or bone turnover and levels of IL-6 and TNF did not differ between those with and without reduced bone density [24]. However, they found that low body mass index (BMI), increased bone turnover, and low IGF-1 were independent predictors of low spinal bone density. Despite prevalent elevations of TNF*α* and IL-6, levels did not correlate with degree of bone loss. Similarly, in another study in which the osteoporosis percentages did not significantly differ between viral cirrhotics and controls, although the serum levels of sTNFR were elevated, the researchers did not find any relationship between this cytokine receptor level and bone mass [25]. Although this study support our findings in which the osteopenia prevalences was not different from controls, they explain the possibility of absence of negative effect of TNF on bone mass as the elevated serum levels of both osteoprotegerin and serum estradiol on bone of their female patient population. 

In a study conducted by Goral et al., IL-6 and TNF-*α* were found to be elevated in cirrhotic patients with osteopenia compared to controls [26]. However, the mean age of their cirrhotic patient population is about a decade older than controls. Probably when corrected for ages, maybe, Goral et al. would not find any difference between their older cirrhotic patients and younger controls. Similarly, Antonio Díez-Ruiz et al. suggested that the decrease in bone mass in their alcoholic cirrhotic patients could be related to elevated serum levels of TNF-alpha and IL-2 [27]. On the other hand, in both of these two studies, serum osteocalcin levels as well as the IGF-1 were also found to be reduced. So, it is difficult to interpret from the results of these studies whether elevated serum levels of cytokines contribute to bone loss in cirrhotic patients.

Although our patients did not have a higher frequency of osteoporosis compared to controls, we demonstrated that osteoblastic activity was in a decreasing state. In this study, the level of osteocalcin was found to be low in viral and alcoholic cirrhotic patients, as well as in chronic alcoholic patients without cirrhosis. This finding suggested that osteoblastic activity did not decrease only in cases with liver disease, but also in chronic alcoholics without liver disease probably due to the direct toxic effect of ethanol osteoblasts [28, 29]. In our study, we could not detect any decrease in 25-OH vitamin D serum levels in cirrhotic patients with or without osteopenia. The normal values of serum vitamin D, phosphorus, and 24-hour urinary calcium and phosphorus levels in our patients suggested that the decreased serum calcium levels were mostly related to decreased albumin levels. 

The smaller number of cirrhotic patients in our study compared to other studies was considered as a limitation of our study. In addition, collection of cross-sectional instead of longitudinal cytokine levels may also be misleading. However, selection of only male patients and the presence of a low prevalence of osteoporosis in cirrhotic patients included in the study irrespective of the disease stage demonstrated that the incidence of hepatic osteodystrophy was lower than what was previously anticipated. We also concluded that the serum cytokines IL-1, IL-2, IL-6, IL-8, and TNF-*α* did not play a role in the pathogenesis of hepatic osteodystrophy.

## Figures and Tables

**Table 1:** Basic demographic and laboratory data* of the study population.

Parameter	Control	Cirrhosis	Cirrhosis-viral	Cirrhosis-alcoholic	Alcoholic
Number of patients (male)	17	44	27	17	15
Etiology (HBV/HCV)	—	—	20/7	—	—
Age	48.7 ± 3.8	50.8 ± 8.8	49.6 ± 9.3	50.7 ± 7.7	46.9 ± 11.0
BMI (kg/m^2^)	28.1 ± 3.9	26.97 ± 2.79	26.47 ± 2.47	27.78 ± 3.16	25.37 ± 3.49
Alcohol (g/day)	—	—	—	111 ± 60	122 ± 50
Cigarette	3/17^†^	38/44	21/27	13/17	15/15
Food	16/17	43/44	26/27	17/17	15/15
Caffeine	7/17	11/44	8/27	3/17^†^	11/15
AST	24 ± 2	65 ± 40	69 ± 40^‡^	59 ± 40^‡^	28 ± 36
ALP	63 ± 21	109 ± 69	84 ± 36	147 ± 90^¶^	96 ± 42
GGT	41 ± 12	85 ± 82	59 ± 47	126 ± 108^§^	117 ± 140^§^
Total bilirubin	1.1 ± 0.2	2.3 ± 1.9	2.2 ± 1.8^†^	2.6 ± 2.2^†^	0.9 ± 0.3
Total protein	6.7 ± 0.1	7.1 ± 2.7	6.8 ± 0.7	7.6 ± 4.3	6.5 ± 0.6
Albumin	4.2 ± 0.2	2.7 ± 0.8	2.8 ± 0.9^†^	2.5 ± 0.7^†^	3.7 ± 0.5
PT	12.8 ± 0.9	16.9 ± 2.8	17.7 ± 3.4^†^	17.0 ± 2.1^†^	12.3 ± 09
Hemoglobin	15.2 ± 0.7	12.1 ± 2.4	12.6 ± 2.6^†^	11.1 ± 1.8^†^	15.7 ± 1.2
Platelet	227000 ± 26000	132000 ± 65000^a^	126000 ± 64000^†^	142000 ± 69000^†^	223000 ± 66000
WBC	6400 ± 500	4146 ± 2229	4800 ± 1998^†^	3735 ± 2510^†^	8626 ± 1627

*Data are presented as mean ± standard deviation. ^†^ *P* < 0.001 versus normal, ^‡^ *P* < 0.001 versus normal, ^¶^ *P* < 0.001 versus all other groups, ^§^ *P* < 0.001 versus control and viral cirrhosis.

**Table 2:** Lumbar and femur T-scores, cytokines, hormones and bone metabolism data* of the study population.

Parameter	Control	Cirrhosis	Cirrhosis-viral	Cirrhosis-alcoholic	Alcoholic
Lumbar T score	−0.16 ± 0.97	−0.29 ± 1.04	−0.15 ± 0.97	−0.50 ± 1.12	−0.61 ± 1.54
Femur T Score	0.18 ± 1.38	0.27 ± 1.07	0.35 ± 1.04	0.14 ± 1.13	0.13 ± 1.29
Interleukin 1 (ng/mL)	15.1 ± 55.7	7.6 ± 28.3	11.1 ± 35.8	1.9 ± 2.9	2.0 ± 3.2
Interleukin 2 (ng/mL)	1435 ± 419	2623 ± 875^†^	2428 ± 637^†^	2931 ± 1111^†^	1746 ± 458
Interleukin 6 (ng/mL)	49.6 ± 123.2	73.9 ± 106.0^†^	76.1 ± 129.7^†^	70.3 ± 54.8^†^	43.7 ± 72.6
Interleukin 8 (ng/ml)	106.5 ± 148.6	164.4 ± 169.3	195.8 ± 203.2	114.6 ± 75.0	93.3 ± 79.5
TNF-alpha (ng/mL)	33.4 ± 193.0	−6.3 ± 62.9	3.2 ± 78.7	−21.3 ± 14.0	−27.4 ± 10.1
Vitamin D	16.9 ± 17.3	23 ± 30	16.2 ± 21.0	34.9 ± 38.5	29 ± 26.5
Total testosterone	434.7 ± 147.4	462 ± 268	474 ± 277	442 ± 260	688 ± 204^‡^
Parathormone	38.6 ± 7.7	49.0 ± 25.7	49.9 ± 28.9	47.7 ± 20.6	47.0 ± 15.1
Osteocalcin (*μ*g/L)	14.8 ± 5.5^¶^	4.2 ± 2.8	4.6 ± 3.0	3.6 ± 2.3	7.9 ± 5.7
Calcitonin	9.0 ± 2.0	6.3 ± 3.1	6.0 ± 2.6	6.9 ± 3.9	6.3 ± 2.8
Calcium	9.3 ± 0.3	8.3 ± 0.6	8.3 ± 0.7^§^	8.3 ± 0.6^§^	9.0 ± 0.4
Phosphorus	2.9 ± 0.4	3.1 ± 0.8	2.9 ± 0.7	3.5 ± 0.8	3.2 ± 0.7
Urinary calcium	89.2 ± 57.7	165 ± 100	162 ± 96	172 ± 109	130 ± 83
Urinary phosphorus	757 ± 873	649 ± 432	629 ± 435	681 ± 440	533 ± 384
T3	3.2 ± 0.7	2.4 ± 0.7	2.5 ± 0.7	2.3 ± 0.6^z^	2.8 ± 0.7
T4	1.5 ± 0.3	1.2 ± 0.3	1.2 ± 0.3	1.3 ± 0.3	1.4 ± 0.2
TSH	1.5 ± 0.9	1.2 ± 0.8	1.0 ± 0.6^ß^	1.4 ± 1.0^ ß^	1.8 ± 0.9

*Data are presented as mean ± standard deviation. ^†^ *P* < 0.001 versus controls, ^‡^ *P* < 0.01 versus controls and cirrhosis, ^¶^ *P* < 0.001 versus all others, ^§^ *P* < 0.001 versus group 1 and 2, ^z^ *P* < 0.05 versus all others, ^ß^ *P* < 0.05 versus controls and alcoholics.
